# Intervention with institutional caregivers for the purpose of supportive education for disadvantaged children: 3C

**DOI:** 10.3389/fpsyg.2024.1493269

**Published:** 2024-11-07

**Authors:** Zeynep Dere, Duygu Akagündüz Eğrikılınç

**Affiliations:** ^1^Department of Child Development, Ege University, Izmir, Türkiye; ^2^Ministry of Family and Social Services, Izmir, Türkiye; ^3^Selcuk University, Konya, Türkiye

**Keywords:** child welfare, child care, supportive education, intervention, communication and interaction

## Abstract

**Introduction:**

Children in institutional care may have limited access to educational opportunities compared to children in parental care. Improving and enriching the educational practices of these children is important for their well-being. In this study, the Child Development, Communication and Care (3C) Training Program was prepared by the researchers. The 3C education approach emphasizes early childhood development and education. Particular focus is on the use of communication techniques in the education of young children. Our goal with 3C is to increase caregivers’ knowledge of child development and support them to communicate effectively with children. Through this curriculum framework, children are encouraged to interact effectively and empathically with others. Therefore, the curriculum framework emphasizes understanding emotions and building trusting relationships as key components in the holistic development of young disadvantaged children.

**Methods:**

A quasi-experimental design with pre-test-post-control group was used in the study. 16 sessions of 3C were applied to the personnel in the experimental group via distance education. Data were collected from the participants before and after the start of the training program by using the Child Education Competence Scale-Parent Form, Personal Information Form, and Training Evaluation Questionnaire.

**Results:**

As a result, significant differences were obtained between the experimental and control groups in favor of the group receiving training (z = 3.57, p<0.05). Accordingly, it can be said that 3C contributes to the holistic development of the child.

**Discussion:**

It is hoped that such work could enhance the developmental outcomes of young children in institutional care and improve the quality of early childhood education.

## 1 Introduction

The primary goal of all educational research is to promote the best interests of children. Not all children have the same developmental opportunities, and in such cases, the state and society have several responsibilities in the care, upbringing and education of children (Gatcho et al., 2024). Children who can be considered disadvantaged are taken into institutional care under the protection law. Considering the developmental characteristics of these children, their educational needs should be developed with different support programs. Adults play the role of educators in children’s lives, whether in institutions or with their families. For this reason, the indirect effects of adult education can be seen on children. 3C is an intervention program created with alternative educational content for this need.

The 3C education approach emphasizes early childhood development and education. Particular focus is on the use of communication techniques in the education of young children and the use of effective communication methods. Good communication can be the key to resolving conflicts. Communicating with young children in particular can sometimes be difficult for adults. For this, it is necessary to know child development and the stages of development well. Various interventions are mentioned in the literature to improve the development of children staying in institutions. While some studies draw attention to caregiver training, some studies focus on providing additional stimuli to children in institutions. Our goal with 3C is to increase caregivers’ knowledge of child development and support them to communicate effectively with children.

A child’s first education starts in the family. The personnel working in institutions are the ones who serve as educators in the lives of children with family deprivation. In the ecological systems theory developed by Urie Bronfenbrenner, the factors surrounding the child and affecting his/her development are discussed in layers from the inside out according to their effects on the child’s development (Bronfenbrenner, 1994). The environment is divided into four layers: microsystem, mesosystem, exosystem and macrosystem. The microsystem is the closest environment with which the child has a one-to-one relationship. The mesosystem is the relationships between microsystems. The exosystem is the systems that affect the individuals who play a role in the child’s development. And the macrosystem is the most external layer of the theory, which includes cultural and ideological factors (Bronfenbrenner, 1979). Accordingly, caregivers are the ones who establish a one-to-one relationship with the child in the microsystem. According to Bowlby (1969), the warm and sincere relationship established with the caregiver in the early period significantly affects the holistic development of the child. It can be said that a healthy attachment relationship between the caregiver and the child will improve the child’s sense of security in life. Behavioral patterns developed by institutionalized children based on their close relationships with caregivers instead of their parents, who are their attachment figures, shape their mental schemas and affect their expectations, beliefs and attitudes in close relationships in the following years. In line with these theories, it is thought that the training of care personnel gains importance (Morais et al., 2023).

Education for children at risk helps to maximize their potential. The impact of disadvantage on children can also be reduced in early childhood (NAO, 2024). Research has found that institutions mostly prioritize needs such as health, hygiene and nutrition. There is a prevailing view that educational content is limited. The focus was on the staff working in institutions and changes in institutional structures. The view that the developmental delays observed in children in institutions can be minimized with educational interventions that encourage children’s relationships with adults has come to the fore (Zeanah et al., 2003; Muhamedrahimov et al., 2004; Hadley et al., 2024).

It is possible to say that the quality of care provided is very important for children’s psychology, development and education. It has a significant impact on children’s development and low academic performance, especially for children who have been subjected to maltreatment, children with a history of trauma, and children experiencing maternal and paternal deprivation. The insufficient number of staff in the institution providing care services, the lack of adequate training equipment for the staff, and the excessive workload damage the educational life of children (Hermenau et al., 2017; INEE, 2024). An association has been established between adverse childhood experiences and negative outcomes in cognitive and emotional functioning. It is discussed that the absence of parents, especially in the early years of life, can have detrimental effects on cognitive abilities (Sadeghzadeh and Bagheri, 2023). In the development, education and welfare of the child, the importance of communication and interaction with the people around him/her gains value (Lery et al., 2024). For this reason, there is a need to investigate the effectiveness of evidence-based training programs to support institutional organizations by offering them to caregiver (Damery et al., 2021; Do and McCoy, 2024).

Just as the gains of children increase when families consciously support their children’s education, it is necessary to support care personnel when it is considered that care personnel in institutions provide similar support to that provided by the family to children who grow up away from the family environment UNICEF (2024a). It is necessary to contribute to the development and educational life of children under protection and care through studies aimed at these personnel.

According to UNICEF (2024b), nearly half a million children across Europe and Central Asia today live in residential care facilities, including large-scale institutions. The rate of children living in residential care facilities across Europe and Central Asia is double the global average, with 232 per 100.000 children living in residential care facilities compared to 105 per 100.000 globally. Children growing up in institutional care spend most of their time in the institution and with the caregivers. Therefore, it is important to focus on the 3C training program to ensure that caregivers are knowledgeable about early childhood development and education. In this study, it was aimed to support the caregivers of children in the institution with the Child Development, Communication and Care (3C) Training Program prepared by the researchers.

The curriculum framework is an educational enrichment for children’s learning. Through this curriculum framework, children are encouraged to interact effectively and empathically with others. Therefore, the curriculum framework emphasizes understanding emotions and building trusting relationships as key components in the holistic development of young disadvantaged children. In this way, it may be easier to find solutions to the behavioral and emotional problems seen in children staying in institutions. In addition, this educational intervention sets an example for social work studies in Turkey. The more disadvantaged populations are supported by educational interventions, the more it is hoped that nationwide welfare will increase. At the same time, presenting the 3Cs through the distance education model provided time, space and economic advantages to the participants. From this perspective, it can be said that 3C is a first in Turkey.

A changing world leads to changing needs, and the developmental characteristics and needs of each generation also differ. Caregivers, who are employees of the institution, are also likely to need support and assistance for various reasons. All individuals may need support both emotionally and in practice to raise children (Damery et al., 2021). Although it is thought that the staff working in the field have knowledge and experience about child development and education, it is assumed that updating the information, gaining awareness about new approaches and sharing case examples through training based on group interaction will give people the opportunity to increase their knowledge. In addition, as their skills in child rearing improve, they will be more sensitive to the developmental and educational needs of children (Do and McCoy, 2024).

In this study, the effect of the 3C Training Program given to the caregiver responsible for the care services of children aged 0–6 years under protection and care was examined. For this purpose, answers to the following questions were sought;

1. Does the implemented 3C Training Program differ according to various variables of caregivers?
2. Is the 3C Training Program effective for the participants in terms of the following items;
  - Guiding the child,
  - Child development,
  - Getting to know the child,
  - Communication/Interaction,
  - In the sub-dimensions of gaining responsibility
3. Is 3C an effective intervention program for caregivers?

## 2 Materials and methods

In this section of the article, the research model, procedure, study group, data collection tools and data analysis are presented.

In this study, the target group is the caregiver providing care services to children in the institution. Within the scope of the research, the training program prepared for the caregivers was presented to the participants through distance education method. Web-based trainings are more flexible and interactive for adult education compared to traditional trainings and therefore have become preferable alternatives for education. In distance education, study resources can be accessed instantly via the internet with a wide variety of presentation methods (Dogu et al., 2016; Gluoksnyte, 2022).

### 2.1 Research model

A quasi-experimental design with pre-test - post-test control group was used in the study. Quasi-experimental studies are studies to determine the effect of the differences determined by the researchers on the dependent variable. These methods are frequently encountered in educational research. Quasi-experimental designs are a good alternative for situations where groups cannot be randomly assigned and are predetermined (Baştürk, 2014). In this study, the experimental and control groups were formed on a voluntary basis, and care was taken to ensure that the participants of the experimental group consisted of maintenance personnel who could fulfill the requirements of the training, had the technological equipment to follow distance education and were nominated by the relevant institution. For this reason, it can be said that the study group was formed by the purposive sampling method, which is one of the non-probability sampling techniques.

In the study, in order to measure the effect of the training given to the experimental group, an online pre-test-post-test was applied to the caregivers. The independent variable of the research design is the “Child Development, Communication and Care (3C) Training Program” applied to the caregivers. The dependent variable is “Child Education Competence.” To examine the effect of the training program, a control group was formed; the participants in the control group were not given any training during the study and were only asked to fill in the Personal Information Form and the Child Education Competence Scale. At the end of the study, the same training was also given to the caregivers determined as the control group, and it was ensured that all caregivers benefited from the training program.

### 2.2 Procedure

Before starting the study, permission was obtained from Ege University Social and Human Sciences Scientific Research and Publication Ethics Committee with the decision dated 29.06.2022 and numbered 1.518. After the Ethics Committee permission was obtained, official correspondence was made with the relevant institutions so that the study could be conducted with the people working as caregivers in the Children’s Homes Site affiliated to the İzmir Provincial Directorate of Family and Social Services. Due to working hours, the institutions agreed to conduct the training in the form of distance education. As a result, approval was obtained for the research with the “Approval” of the Ministry of Family and Social Services, Education and Publication Department dated 14.11.2022 and numbered 429. After the institutional approvals were completed, a research project was applied to Ege University Scientific Research Projects Coordinatorship to include the current research within the scope of a project and to carry out a distance education program through Ege University Continuing Education Center. It was accepted as a scientific research project titled “Investigating the Effects of the Education Program Implemented on Child Care Providers” with the project number 28496, supported and financed by Ege University. Following the approval of the project, the relevant organization was contacted and a working group was formed with the participation of 45 maintenance personnel.

Before starting the training, the participants were given a “Personal Information Form” and the “Child Education Competence Scale” for which permission was obtained. The collection of the pretests took 2 days in total. The participants were informed that the training would be asynchronous. Those who completed the training were checked from the EGESEM system. The posttest was applied to the participants who completed the training for 1 day and they were thanked. The posttest data of the control group was collected face to face for 1 day.

To determine whether the data set was normally distributed, a normality test was performed using the SPSS 22 program, and after the extreme values were removed, it was observed that the data showed a normal distribution and the number of participants was determined as 43. The participants were divided into two groups as experimental group consisting of 23 participants and control group consisting of 20 participants. Participants in the experimental group were given access to the training program titled “Child Development, Communication and Care (3C)” consisting of 16 sessions by defining a username and password through Ege University Continuing Education Center. Each session of the program lasts approximately 20 min. Participants who completed the sessions completely received a course completion certificate approved by Ege University and e-government. At the end of the training, the participants were asked to fill out the “Child Education Competence Scale” as a post-test and also the “Training Evaluation Questionnaire.” After the post-tests were collected from the experimental group and the study was completed, the same training was given to the caregivers in the control group under the same conditions and all caregivers in the institution benefited from the training program.

In addition, the 16-session training program prepared for the 3C intervention program developed within the scope of the research was made available free of charge through Ege University Continuing Education Center (the other two sessions were not conducted because they were pre-test-post-test applications applied to the study group). It was accepted as a Social Responsibility Project as a “Child Development Education Certificate Program” with the number 23SSP0550002 at Ege University. Within the scope of the Social Responsibility Project, university students, child developers and other professionals who want to improve themselves in child development and education were trained. In this way, nearly 1.500 e-government-approved “Child Development Education Certificate Program” certificates were issued through Ege University Continuing Education Center.

### 2.3 Study group

This project focused on a study group of 43 caregivers. The participants were divided into two groups: an experimental group of 23 participants and a control group of 20 participants. A 16-session 3C intervention program was applied to the caregivers in the experimental group, while no intervention was applied to the control group.

### 2.4 Data collection tools

#### 2.4.1 Personal information form

The demographic information of the caregivers participating in the study was collected online/face-to-face with the Personal Information Form prepared by the researchers. The personal information form included open and multiple-choice questions to determine information about the age, educational status, marital status of the caregivers and questions about their expectations from education and the groups of children they work with.

#### 2.4.2 Child education competence scale (CECS)

The scale was developed by Yeşil et al. (2018) to determine the competency levels of adults in child education based on self-assessment. The Cronbach alpha reliability coefficient of the 5-factor, 5-point Likert-type scale containing 37 items is 0.922. The scale has sub-dimensions such as Guiding the Child, Developing the Child, Getting to Know the Child, Communication/Interaction, Gaining Responsibility and includes statements such as “I can make my child share his/her problems with me easily, I can make my child behave by the rules.” The raw scores obtained from the scale are interpreted by converting them into standard scores and there are statements about what the value range means. 20–35 points are defined as “Very Inadequate,” 36–51 points as “Inadequate,” 52–67 points as “Partially Adequate,” 68–83 points as “Adequate” and 84–100 points as “Very Adequate.” Each of the items in the factors is graded as “(1) Never, (2) Rarely, (3) Sometimes, (4) Most of the time, and (5) Always” reflecting the level of the participants’ indication of how often they can do it. A parent who can “always” perform the skill in the relevant item is considered to be “very competent” in terms of that skill, while a parent who can “never” perform the skill is considered to be “very inadequate.” As a result of the analysis of the scale, it was found to be a valid and reliable tool that can be used for families (Yeşil et al., 2018).

As a matter of academic courtesy, permission for use was obtained from the researchers via e-mail.

#### 2.4.3 Child development, communication and care education program (3C)

Within the scope of the research, a sixteen-session distance education program called Child Development, Communication and Care (3C) was designed through Ege University Continuing Education Center in order not to affect the working hours of the caregivers. While creating the training program, the relevant institution was consulted and the topics for the needs of the caregivers were determined.

Samples session from the program is presented below;

Example 1

1.1. Session Topic: What is communication? What are communication methods?

1.2. Session model and method: Asynchronous distance education, presentation.

1.3. Session Objective: What is communication? To gain competencies related to communication types and techniques between individuals, communication barriers, healthy communication.

1.4. Session Outcomes: The person knows what communication is. The person learns the types of communication between individuals. Learns about interpersonal communication techniques. Lists communication barriers. Knows the elements of healthy communication. Examples of positive and negative communication techniques. Decides on appropriate ways to solve interpersonal problems.

1.5. Session Content: Communication. The importance of communication. Communication tools. Communication types and techniques. Listening skills. Reacting. The concept of empathy. Empathy stages. Positive communication. Negative communication. Communication Barriers. Interpersonal communication. Factors preventing interpersonal communication. Interpersonal communication problems. Using appropriate ways of interpersonal problem-solving.

Example 2

2.1. Session Topic: Cognitive development in early childhood.

2.2. Session model and method: Asynchronous distance education, presentation.

2.3. Session Objective: To analyze the characteristics of cognitive development in early childhood. Discussing the concept of cognitive development in early childhood.

2.4. Session Outcomes: Knows the concepts of cognitive development in early childhood. Knows the stages of cognitive development in early childhood. Learns Piaget’s theory of cognitive development. Understands the relationship between cognitive development and other developmental areas.

2.5. Session Content: Piaget’s theory of cognitive development. Cognitive development concepts. The impact of cognitive development on other developmental areas. Cognitive development stages in early childhood. Pre-operational period. Cognitive development indicators in early childhood.

Example 3

3.1. Session Topic: Care, nutrition and hygiene of young children.

3.2. Session model and method: Asynchronous distance education, presentation.

3.3. Session Objective: Learning about baby nutrition, bottle use, hygiene and hygiene education.

3.4. Session Outcomes: The person knows about baby nutrition. The person learns about complementary foods and supplementary foods. Learns about bottle use and cleaning. The person has knowledge about providing their own hygiene. Knows the elements of providing baby hygiene.

3.5. Session Content: Baby nutrition, supplementary food, complementary foods, bottle use and cleaning, personal hygiene, how should baby hygiene be provided? How should hygiene education be given to the child?

### 2.5 Data analysis

In the experimental part of the study, the raw scores of the participants from the Child Education Competence Scale both before and after the training were converted into standard scores. Then, according to the normal distribution of the data by looking at the *p*-value and kurtosis-skewness values in the Shapiro–Wilk Normality test to determine whether the data set was normally distributed (Hair et al., 2013), independent samples t-test, Wilcoxon Signed Ranks Test and ANOVA were used to test whether there was a significant difference between the groups. All tests were performed using SPSS.

## 3 Results

In this part of the study, the findings obtained as a result of the analysis of the data obtained from the measurements made before and after the training are presented in tables and figures.

First of all, the first sub-problem of the study, “Does the 3C Training Program differ according to various variables of the caregivers?” was sought to be answered. Whether the scores of the participants from the CDE differed according to some variables was analyzed using One-Way Analysis of Variance (ANOVA) and *t*-test according to the number of groups and the results are given in Table 1.

**Table 1:** Mean total scores obtained from the Child Education Competence Scale according to some variables of caregivers and analysis results

**Variables**	**Groups**	*n*	**B. E.**		**A. E.**	
			*X_*	*p*	*X_*	*p*
Age	Age between 20-30	6	72.97	0.022*	74.95	0.113
	Age between 30-40**	24	80.56		84.90	
	Over 40	13	76.17		83.03	
	Total	43	78.17		82.95	
Education Status	High School	21	77.76	0.616	80.46	0.297
	Associate Degree	18	79.22		84.92	
	Bachelor’s Degree	4	75.67		87.16	
	Total	43	78.17		82.95	
Type of Education	Vocational High School	26	76.79	0.380	81.12	0.305
	Public Education	10	80.97		83.78	
	Formal Education at University	2	77.02		82.97	
	Open Education	5	80.21		90.81	
	Total	43	78.17		82.95	
Duration of Employment	1 to 5 years**	3	81.08	0.761	96.94	0.043*
	5 to 10 years	21	78.01		82.83	
	10 years and more	19	77.89		80.88	
	Total	43	78.17		82.95	
Age Group worked with	0 to 1 year old	13	78.04	0.348	81.29	0.839
	2 to 3 years old	4	78.51		80.81	
	3 to 4 years old	9	74.77		84.86	
	5 to 6 years old	17	80.00		83.71	
	Total	43	78.17		82.95	
Marital Status	Married	37	78.39	0.618	82.58	0.675
	Single	6	76.84		85.22	
Childbearing Status	Yes	36	78.25	0.864	82.37	0.415
	No	7	77.76		85.94	

When Table 1 is examined, it is seen that the total scores of the participants from the scale before the 3C training show a significant difference only according to age and after the training according to the working period. In the TUKEY HSD Test conducted to determine from which age group this age-related difference originated, it was determined that the difference between the participants between the ages of 20–30 and 30–40 was significant. It was thought that the older participants scored higher on the Child Education Competence Scale, and this age-related difference may be due to their life experiences in raising children.

When we look at the post-training period, it is understood that the averages between the groups differ in terms of working time. It was found that the mean scores on the Child Education Competence Scale showed a significant difference in favor of the participants who worked between 1 to 5 years. This difference in terms of working time may be related to the stress, professional satisfaction or wear and tear of working life. Looking at Table 1, it is possible to say that the scores of the child education competence scale are in favor of the caregivers who have just started their professional life.

The second sub-problem of the study, “Is the 3C Education Program effective for the participants in the sub-dimensions of guiding the child, developing the child, getting to know the child, communication/interaction, and gaining responsibility?” was sought to be answered. The results are presented in Table 2.

**Table 2:** *T*-test results of the scores of the caregivers in the experimental and control groups before and after the 3C training program in the sub-dimensions of the Child Education Competence Scale.

CECS subscales	Groups	*n*	*X_*	*t*	*p*
Before 3C					
Guiding the child	Experimental	23	11,217	−1,075	0.290*
	Control	20	10,450		
Developing the child	Experimental	23	15,478	−1,215	0.231*
	Control	20	14,750		
Getting to know the child	Experimental	23	33,261	−4,241	0.000
	Control	20	29,100		
Communication/interaction	Experimental	23	47,609	−4,677	0.000
	Control	20	41,650		
Gaining responsibility	Experimental	23	44,652	−4,992	0.000
	Control	20	40,350		
After 3C					
Guiding the child	Experimental	23	13,739	−7,206	0.000**
	Control	20	10,450		
Developing the child	Experimental	23	17,826	−6,363	0.000**
	Control	20	14,750		
Getting to know the child	Experimental	23	29,100	−8,849	0.000
	Control	20	29,100		
Communication/interaction	Experimental	23	51,174	−8,227	0.000
	Control	20	41,650		
Gaining responsibility	Experimental	23	48,826	−7,768	0.000
	Control	20	40,350		

*No significant difference before training.

**A significant difference was found after the training.

When Table 2 is examined, it can be said that the mean scores of the participants in the experimental group before the training increased after the training, so it can be said that the training program contributed to the participants’ skills in child education. At the same time, the difference between the mean scores of the control and experimental groups in all sub-dimensions after the training was significant (*p*<0.05). In addition, while there was no significant difference between the groups in the “Guiding the Child” and “Developing the Child” sub-dimensions of the Child Education Competence Scale before the training (*p*>0.05); it was found that there was a significant difference between the groups in these sub-dimensions after the training (*p*<0.05).

It can be said that the caregivers in the experimental group were more advantageous than the caregivers in the control group in the sub-dimensions of recognizing the child, communication with the child, interaction and giving responsibility to the child (*p*<0.05).

The Wilcoxon Signed Ranks Test was used to analyze whether the difference between the mean scores of the experimental group from the Child Education Competence Scale before and after the training was significant.

It was observed that the difference between the average scores of the experimental groups at the beginning and after the training was significant for all sub-dimensions (*p*<0.05).

It can be said that 3C, which was prepared for caregivers, is a training program that has positive effects on caregivers who receive training in supporting the sub-dimensions of child guidance, child development, getting to know children, communication with children, and giving children responsibility (*p*<0.05).

According to these results, 3C was effective in improving the skills of caregivers in contributing to the child’s holistic development in the areas of guiding the child, supporting the child’s development, recognizing the child, communicating effectively with the child and giving them responsibility.

The answer to the third sub-problem of the study, “Is the 3C training program effective for caregivers?” is presented in Table 3. To examine whether it is an effective program for caregivers, analysis was performed before and after 3C.

**Table 3:** Mean scores obtained from the Child Education Competence Scale.

		*n*	*X_*	SS
Before 3C	Experimental group	23	82.2748	4.45018
	Control group	20	73.4555	6.05659
After 3C	Experimental group	23	91.2048	4.45018
	Control group	20	73.4555	6.22914

When Table 3 is examined, it is seen that the mean score of the control group was $\bar{X}$ =73.45 before the 3C training program and remained constant as $\bar{X}$ =73.45 after the training program. On the other hand, before the 3C training program, the mean score of the experimental group obtained from the Child Education Competence Scale was $\bar{X}$ =82.27, and after the training program, the mean score of the experimental group increased to $\bar{X}$ =91.20.

Accordingly, Wilcoxon Signed Ranks Test was used to analyze whether the difference between the mean scores of the experimental group was significant.

As a result, it was found that there was a significant difference between the Child Education Competency Scale scores of the participants in the experimental group (*z* = 3.57, *p*<0.05). Considering the rank averages and the sum of the difference scores, it was understood that this difference was in favor of positive ranks, that is, the post-test score. The 3C training program was effective in teaching caregivers how to communicate with the child in the holistic development of the child. In addition, it effectively supported the child in terms of child recognition techniques, communication techniques, guiding the child and taking responsibility. This suggests that 3C has a positive effect on caregivers in child development and education.

## 4 Discussion

In this study, the average age of caregivers working in child care institutions is 36. The majority of caregivers are married and have children. Looking at the literature, it was observed that the care caregivers working in the Ministry of Family and Social Services were between the ages of 30–40 and were married and had children (Tambulut and Eker, 2019; Aslan and Erbay, 2017). This finding in the literature is similar to the findings obtained in this study.

When the level of education of the maintenance personnel is taken into consideration, it is seen that 22 of them are high school graduates and 23 of them have an associate degree or bachelor’s degree. Considering that the level of education of the maintenance personnel must be at least a high school graduate according to the relevant regulation, it can be said that this finding is compatible with the current situation (Official Gazette, 2014).

In this study, when the relationship between the demographic variables of all participants before the 3C training program and the total scores they received from the Child Education Competence Scale is examined, it is seen that the difference between the scores obtained from the scale is significant as age increases. In the study conducted by Tambulut and Eker (2019) with care personnel, it was observed that as age increased, education level, income level and depression scores decreased. It was thought that this age-related difference may be due to the life experiences of the participants in raising children.

Hermenau et al. (2017), investigated the effects of structural interventions in the functioning of institutions and training programs for caregivers on child development. Twenty-four training programs were included in the review. It was reported that the intervention programs applied to caregivers had benefits on children’s social–emotional and cognitive development. The content of 3C provides detailed information on the social–emotional and cognitive development of infants and children. It can be said that the training program implemented in this study has indirect positive effects on the development of disadvantaged children staying in a child home.

Maclean (2003), focused on Romanian children living in both Romania and Canada. She examined children adopted from orphanages for 10 years. According to the findings of the study, the development of children in institutional care was negatively affected in cognitive, physical and social–emotional domains. The longer the period of stay in institutional care, the more the development of children is negatively affected. Considering that 3C includes sessions that support children’s cognitive, physical, social–emotional and language development, it is possible to say that it is an intervention program for areas with developmental negativities.

In another study, it was found that there was a significant difference in the social, language, self-care, cognitive and physical development areas of children in the groups of caregivers who received training after the Portage Early Education Program applied to caregivers (Biber and Ural, 2012). In the current study, a significant difference was also found in the competencies of the caregivers in the experimental group in child development and education according to the Child Education Competency Scale.

Van IJzendoorn et al. (2011), conducted a study with children in institutional care. It was determined that care institutions generally have problems due to “structural neglect,” and these structural neglects were listed as minimum physical conditions, insufficient number of staff, irregular staff shifts, and reasons that reduce the quality of interactions and communication between caregivers and children. The content of 3C includes sessions to support communication skills with the child. In this respect, it is possible to say that 3C effectively supports the communication skills of children in institutional care and caregivers.

Maundeni and Malinga-Musamba (2013), investigated the roles, challenges and needs of caregivers in the welfare of children in institutional care and how they can be strengthened. It was found that caregivers play very important roles in the lives of children. In the process of caregiving, it was found that there are circumstances that make it difficult for caregivers to fulfill their responsibilities and roles. Sometimes children in institutional care experience stress. As a result of the stress, children have difficulty adapting to the loss of their parents and may have problems with caregivers. 3C is a training program that provides information on how institutional staff can communicate with children and what the communication barriers are.

In their paper, Wright et al. (2014), present a case study of a project to improve the health, safety and development of children who were brought from birth to a large orphanage in Nepal and cared for until the age of 6. There were two interventions in the project. The first was to improve the physical infrastructure. The second intervention was the provision of training and mentoring to staff and support services to caregivers. These interventions resulted in positive outcomes for children’s health and development, including a reduction in communicable diseases and increased social interaction with caregivers. Similar to this research, the content of 3C includes presentations on how to support adult-child communication skills as well as information on hygiene for infants and children in institutional care.

Oliveira et al. (2015), focused on 72 preschool children who stayed in institutions for at least 6 months. In the study, the caregivers working in the institutions conducted an assessment for each child. For the assessment, the Child Behavior Checklist and a measurement tool to determine the attachment status of children were used. The quality of care in the institution was assessed through staff reports and direct observation. As a result of the study, it was found that secure attachment behaviors in children increased when caregivers interacted more with children and exhibited more sensitive and non-discriminatory behaviors. Negatively significant results were obtained between a closer relationship with the caregiver and insecure attachment behavior. Positive communication, interest and developmental knowledge of caregivers support children’s development positively. 3C is an intervention program that provides caregivers with information about early attachment.

Lecannelier et al. (2014), in a study conducted with the Chilean government, targeted 62 infants in a care institution and the staff providing care services to infants. The study, which was planned with a quasi-experimental design, was conducted in two phases. In the first phase, the development of the babies in the institution was evaluated before the intervention was applied to the caregivers. In the second phase, the social and emotional development of the infants was evaluated after the intervention. After the first evaluation, an intervention program was applied to the caregivers. Before the intervention was applied to the caregivers, it was determined that the infants were insecurely attached and had delays in their psycho-motor skills. They were told that insecurely attached infants were likely to be vulnerable in the future. According to the findings of the study, the intervention applied to the caregivers was found to have positive improvements in the social–emotional development of infants. Long-term training of caregivers made positive contributions in all areas of infants’ development. What was striking in the study was the socio-cultural change not only in the infants but also in the caregivers. The caregivers changed their language and communication style positively after the intervention. It is possible to say that 3C is a training program that includes sessions to support communication skills with the child as well as information to support children’s cognitive, physical, motor, social–emotional and language development.

Rygaard (2010), created the content for a free e-learning and organizational development program on providing quality care for orphanages and foster families in the Fair Start project. The aim of the study was to develop free science-based Internet training programs for orphanages and foster families, specifically aimed at improving the care of young children in public custody. Free e-learning development programs were designed in six local languages. Participation in the program should only require internet access. The results showed that the program for caregivers played an important positive role in supporting children’s development. Similarly, the 3C intervention is also an online training program and as a result of the study, the scores of caregivers who received 3C increased after the intervention. These findings are in line with the findings of the current study.

Sparling et al. (2005), conducted an experimental study in order to improve the quality of life and support the social–emotional development of children in a child home. Newborn infants and infants up to 48 months of age were included in the experimental group. An early intervention program including adult-child communication, enriched care and educational activities was created. It was concluded that the intervention program developed within the scope of the research increased institutional quality. Positive improvements were observed in children’s physical growth, cognitive, language, motor and social–emotional skills. The scores obtained from the caregivers in the 3C group were significantly higher in favor of the children as a result of the intervention program. This finding supports the findings of the study.

Muhamedrahimov et al. (2004), implemented an early intervention program in orphanages in St. Petersburg, Russian Federation. The program focused on the analysis of the orphanage system and statistical data on children, caregivers and their interaction. According to the results of the research, it was found that before the intervention there were significant deficiencies in the consistent behavior and emotional reactions of the caregivers. The intervention program has two main elements. The first one is the training of caregivers and structural changes in the orphanage to promote family-like conditions for the children, and the second main element is the implementation of an intervention program. The first element, structural change interventions, can be listed as increasing the stability and consistency of caregivers and creating a more family-like environment to support relationship building. In the content of the intervention program, which is the second element of the study, a “training of trainers” approach was used. For example, caregivers were instructed to respond to children in a warm, caring, respectful and interactive manner. As a result of the research, it was seen that the intervention program applied to the child home staff had positive effects and there was a decrease in the deficiencies identified before the intervention. Similar to the subjects of this study, positive gains were found in favor of the experimental group after the intervention in dimensions such as knowing the child, communication, directing the child and gaining responsibility after the 3C training program.

## 5 Recommendations

Based on the findings obtained as a result of the project, a number of recommendations were developed for researchers. It is possible to list the recommendations for researchers as follows;

- The 3C Program was applied only to the caregivers at Izmir Children’s Homes Site. The 3C Intervention program can be expanded as a TUBITAK project to include all institutions in Turkey,
- 3C Program was conducted online upon the request of the institution. Demonstration and interaction are very important in intervention program-based studies. Future studies can be conducted face-to-face,
- The 3C Program was applied only to personnel providing care services between the ages of 0–6. The Intervention Program can be expanded and the target audience can be structured as care personnel providing services to adolescents.
- It was determined that caregivers benefited from the training. They were eager to improve themselves and contribute to disadvantaged children. Training programs can be prepared in different subjects such as children and art, children and sports, and children’s literature.

## Data Availability

The original contributions presented in the study are included in the article/supplementary material, further inquiries can be directed to the corresponding author.
